# Isolation and identification of *Wickerhamiella tropicalis* from blood culture by MALDI-MS

**DOI:** 10.3389/fcimb.2024.1361432

**Published:** 2024-03-06

**Authors:** Satomi Takei, Kanae Teramoto, Junya Fujimura, Megumi Fujiwara, Mai Suzuki, Yukiko Fukui, Yuji Sekiguchi, Takaaki Kawakami, Masayoshi Chonan, Mitsuru Wakita, Yuki Horiuchi, Takashi Miida, Toshio Naito, Teruo Kirikae, Tatsuya Tada, Yoko Tabe

**Affiliations:** ^1^ Department of Clinical Laboratory Medicine, Juntendo University Graduate School of Medicine, Tokyo, Japan; ^2^ Department of MALDI-TOF MS Practical Application Research, Juntendo University Graduate School of Medicine, Tokyo, Japan; ^3^ Analytical & Measurement Instruments Division, Shimadzu Corporation, Kyoto, Japan; ^4^ Department of Pediatrics, Juntendo University Graduate School of Medicine, Tokyo, Japan; ^5^ Department of General Medicine, Juntendo University Graduate School of Medicine, Tokyo, Japan; ^6^ Biomedical Research Institute, National Institute of Advanced Industrial Science and Technology (AIST), Tsukuba, Ibaraki, Japan; ^7^ Department of Clinical Laboratory, Juntendo University Hospital, Tokyo, Japan; ^8^ Department of Microbiome Research, Juntendo University Graduate School of Medicine, Tokyo, Japan; ^9^ Department of Microbiology, Juntendo University Graduate School of Medicine, Tokyo, Japan

**Keywords:** Wickerhamiella tropicalis, blood culture, yeast, MALDI-MS, whole-genome

## Abstract

*Wickerhamiella* is a genus of budding yeast that is mainly isolated from environmental samples, and 40 species have been detected. The yeast isolated from human clinical samples usually only contain three species: *W. infanticola*, *W. pararugosa* and *W. sorbophila*. In this study, we isolated *W. tropicalis* from a blood sample of a six-year-old female with a history of B-cell precursor lymphoblastic leukemia in Japan in 2022. Though the strain was morphologically identified as *Candida* species by routine microbiological examinations, it was subsequently identified as *W. tropicalis* by sequencing the internal transcribed spacer (ITS) of ribosomal DNA (rDNA). The isolate had amino acid substitutions in ERG11 and FKS1 associated with azole and echinocandin resistance, respectively, in *Candida* species and showed intermediate-resistant to fluconazole and micafungin. The patient was successfully treated with micafungin. Furthermore, matrix-assisted laser desorption/ionization mass spectrometry (MALDI-MS) detected three novel peaks that are specific for *W. tropicalis*, indicating that MALDI-MS analysis is useful for rapid detection of *Wickerhamiella* species in routine microbiological examinations.

## Introduction

Yeast-like fungi and yeasts often cause catheter associated bloodstream infections in immunocompromised patients who receive immunosuppressants, chemotherapies, or organ transplants (Papp et al., 2018). Due to the increase of these therapies, non-*Candida albicans* species have been increasingly identified recently (Kumar et al., 2022). Since these organisms have different susceptibility to antifungal agents, it is critical to identify the species correctly and initiate the appropriate therapies as soon as possible to improve patients’ prognoses.

*Wickerhamiella* is a genus of budding yeast in the family *Trichomonascaceae* that was redescribed in 1973 (van der Walt and Liebenberg, 1973). *Wickerhamiella* species consist of more than 40 species which are mostly environmental organisms. For instance, *W. vanderwaltii* PYCC 3671^T^ (GenBank accession no. GCA_022577775) was isolated from winery equipment in South Africa, *W. sorbophila* DS02^T^ (= NBRC 1583^T^) (GenBank accession no. GCF_002251995) was isolated from industrial water in South Korea in 2015, and *W. spandovensis* PYCC 8431 (GenBank accession no. GCA_022577695) was isolated from alcoholic beverages in Germany. To date, there are only several *Wickerhamiella* species that were isolated from human samples. For example, *W. pararugosa* (*Candida pararugosa*) PH2204 (GenBank accession no. JAMAJR000000000) was isolated from human feces in China in 2019, oral cavity in Italy (Giammanco et al., 2004), and saliva in Japan (Nakagawa et al., 2004). Furthermore, *W. sorbophila* was isolated from human feces in China in 2019 (GenBank accession no. GCA_023629035), and *W. infanticola* from an ear of a baby in Germany in 1995 (GenBank accession no. GCA_004125145). Previous studies have reported that *W. pararugosa* causes bloodstream infections. These species were detected in a six-month-old male with intrauterine growth in Qatar in 2006 (Taj-Aldeen et al., 2014), a three-year-old female with acute lymphoblastic leukemia in Greece in 2008 (Noni et al., 2020), a five-year-old male in Qatar in 2010 (Taj-Aldeen et al., 2014), a 39-year-old female with morbid obesity in the United States in 2017 (El Helou and Palavecino, 2017), and a three-year-old male with acute myeloid leukemia in Iran in 2022 (Nasri et al., 2023). Though *Wickerhamiella tropicalis* was first isolated from environmental samples in 2020 (Sakpuntoon et al., 2020), it has never been isolated from human samples. In this case report, we first isolated *W. tropicalis* in blood cultures that caused severe infections.

Although, gram-staining and fungal isolation media are routinely used to identify yeast-like fungi in clinical laboratories, these methods are insufficient to accurately differentiate fungal species (Pravin Charles et al., 2015). In contrast, genetic sequencing of the internal transcribed spacer (ITS) region of rDNA is an excellent method to accurately differentiate strains. Indeed, predicted taxonomic thresholds for identifying yeast species have been reported to be 98.41% for the ITS-rDNA (Vu et al., 2016). However, sequencing is not suitable for routine microbiological examinations since a short turnaround time is required. Therefore, matrix-assisted laser desorption/ionization mass spectrometry (MALDI-MS) has been developed to identify species quickly and accurately (Eigner et al., 2009). Despite using technology, detection of *W. tropicalis* is currently difficult due to the lack of library database. In this study, we attempted to identify MALDI-MS peaks specific to *Wickerhamiella* species and found three new peaks related to the *Wickerhamiella* species.

### Case report

A six-year-old female who was suffering from B-cell precursor lymphoblastic leukemia was hospitalized from May 2022 at a university hospital for a remission induction therapy. She had repeated fevers and showed high levels of C-reactive protein (CRP) with cervical lymphadenopathies. The treatment with piperacillin/tazobactam (3.0 g/day) and linezolid (450 mg/day) was started and improved her symptoms in June. Though blood cultures were tested 5 times at the time of fever, which were negative, a set of blood culture was positive after 62 h of incubation in July. The isolate was initially identified as *Candida* species. A similar specimen was obtained three days later. A treatment of micafungin (3 mg/kg/day) was started. The blood culture became negative eight days after the treatment.

## Materials and methods

### Fungal strains

A *W. tropicalis* JUWT001 strain was isolated from a patient treated at Juntendo University Hospital in Japan in July 2022. The type strain of *W. tropicalis* TBRC 11426^T^ was obtained from Thailand Bioresource Research Center (TBRC, Thailand) and the type strains of *W. sorbophila* NBRC 1583^T^ and *W. spandovensis* NBRC 10249^T^ (= PYCC 8431^T^) were obtained from the Biological Resource Center, National Institute of Technology and Evaluation (NITE) (Tokyo, Japan). *W. tropicalis* TBRC 11426^T^ was imported into Japan with permission from the Minister of Agriculture, Forestry and Fisheries under the Plant Protection Act. The isolates were cultured in Sabouraud dextrose agar (SDA) plate (Becton, Dickinson-Diagnostic Systems, Sparks, MD, USA), Potato Dextrose Agar (PDA) (Becton, Dickinson-Diagnostic Systems), and/or CHROM agar Candida medium (Kanto Chemical Co., Tokyo, Japan) at 25°C or 35°C under aerobic conditions for 48 hours. The minimum inhibitory concentrations (MICs) of the isolate were determined using the broth microdilution method, as described by the guidelines of the Clinical and Laboratory Standards Institute (CLSI) (CLSI, 2017).

### Drug susceptibility testing

Drug susceptibility was tested by microdilution assay according to the guidelines of the Clinical and Laboratory Standard Institute (CLSI) (CLSI, 2017) using the Yeast-Like Fungus DP (Eiken Chemical Co., Ltd., Tokyo). The ranges of antibiotic concentrations tested were: 5-fluorocytosine 0.12 to 64 μg/mL, amphotericin B 0.03 to 16 μg/mL, fluconazole 0.12 to 64 μg/mL, itraconazole 0.015 to 8 μg/mL, micafungin 0.015 to 16 μg/mL, miconazole 0.03 to 16 μg/mL, and voriconazole 0.015 to 8 μg/mL (**Table 1**).

**Table 1:** Antimicrobial susceptibility profile of *Wickerhamiella tropicalis* JUWT001.

	MICs (μg/mL)
5-fluorocytosine	≤0.12
Amphotericin B	0.12
Fluconazole	4
Itraconazole	0.06
Micafungin	0.5
Miconazole	0.5
Voriconazole	0.12

MICs, the minimum inhibitory concentrations.

### DNA extraction and genome sequencing

DNA of the clinical isolate for sanger sequencing was extracted using DNeasy UltraClean Microbial kit (QIAGEN, Tokyo, Japan). The ITS region of ribosomal DNA (rDNA) was amplified using fungal universal primers Its1 (5’- TCCGTAGGTGAACCTGCGG -3’) and Its4 (5’- TCCTCCGCTTATTGATATGC -3’) (Zhai et al., 2021). The PCR products were sequenced using 3500 XL genetic analyzer (ABI-Hitachi, Tokyo, Japan). Sequence data were analyzed using BLAST on the National Center for Biotechnology Information (NCBI) database.

Genomic DNA of the clinical isolate for whole genome sequencing was extracted as described previously (Mizukoshi et al., 2021). The genome was sequenced by Illumina MiSeq platform using v3 chemistry (600 cycles). Raw reads were trimmed and assembled using CLC Genomic Workbench version 10.0.1 (CLC bio, Aarhus, Denmark). The genome completeness and contamination were assessed using Eukcc v2.1.0 with default settings (Saary et al., 2020). Species identification of the isolate was also determined by ITS using BLAST. Amino acid substitutions in BCR1, BRG1, CBK1, EFG1, and IRE1 (Talapko et al., 2021) associated with virulence, in ERG3, ERG11, PDR1, and TAC1 associated with azole resistance, and in FKS1, FKS2, and GSC1 (Spettel et al., 2019) associated with echinocandin resistance in *Candida* species were analyzed based on whole genome sequencing.

### Phylogenetic analysis

Phylogenetic trees were constructed based on concatenated single-copy marker protein sequences predicted from genomes using GTDB-Tk v2.2.6 software (Chaumeil et al., 2022) and visualized using iTol ver.6. The following type strains were used for a phylogenic tree among *Wickerhamiella* species: *W. alocasiicola* PYCC 8427^T^ (GenBank accession number GCA_022577715), *W. azyma* PYCC 8333^T^ (GCA_022577855), *W.cacticola* NRRL Y-27362^T^ (GCA_003705615), *W. dianesei* PYCC 8330^T^ (GCA_022577725), *W. domercqiae* JCM 9478^T^ (GCA_001599275), *W. galacta* NRRL Y-17645^T^ (GCA_003045245), *W. hasegawae* JCM 12559^T^ (GCA_004125105), *W. infanticola* NRRL Y-17858^T^ (GCA_004125145), *W. kurtzmanii* PYCC 8437^T^ (GCA_022577765), *W. nectarea* PYCC 8436^T^ (GCA_022577815), *W. occidentalis* NRRL Y-27364^T^ (GCA_004125095), *W. pararugosa* PX1910^T^ (GCA_023628975), *W. parazyma* PYCC 8426^T^ (GCA_022577825), *W. sorbophila* NBRC 1583^T^ (GCF_002251995), *W. spandovensis* NBRC 10249^T^ (GCA_022577695), *W. vanderwaltii* PYCC 3671^T^ (GCA_022577775), and *W. versatilis* JCM 5958^T^ (GCA_001600375).

### Accession numbers

The whole-genome sequences of *W. tropicalis* JUWT001 have been deposited in GenBank as accession number DRR488519 (https://www.ncbi.nlm.nih.gov/sra/DRR488519).

### Sample preparation for MALDI-MS

Alpha-cyano-4-hydroxycinnamic acid (CHCA) was used as a matrix. Ten mg of 4-CHCA was dissolved in 1 mL of the solvent consisting of 1% (v/v) trifluoroacetic acid, 35% (v/v) ethanol, 15% (v/v) acetonitrile, and milliQ water. For the analysis using cell lysate, a full loop of fungal cells was dispersed to 200 μL of distilled water in a microtube and mixed with 800 μL of ethanol with zirconia beads. The suspensions were vortexed briefly and centrifuged at 15,000 *g* for 2 min. The pellets were dried for 5 min. After freezing the tubes at -80 °C, the pellets were suspended in 100 μL of 70% formic acid, crushed using a micro tube mixer (MT-400, Tomy Seiko, Tokyo, Japan) for 5 min, suspended in 100 μL of acetonitrile, and centrifuged at 15,000 *g* for 2 min. The analysis using protein fraction was performed as described previously (Teramoto et al., 2019). Briefly, a full loop of fungal cells was dispersed in 500 μL of distilled water in a microtube with zirconia beads. After freezing the tubes at -80 °C, the suspensions were centrifuged at 10,000 *g* for 2 min, 200 μL of supernatants were removed, crushed using a Fast Prep 24 apparatus (Funakoshi Co., Ltd.) for a total of 3 min (9 times for 20 s), and centrifuged at 10,000 *g* for 2 min. The suspensions were washed in milliQ water and centrifuged at 15,000 *g* for 5 min. The supernatants were ultrafiltered using Amicon® Ultra-0.5ml (Merck Millipore, Germany), with cut-off points at 100 kDa, centrifuged at 14,000 *g* for 5 min, and the devices set upside down at 2,000 *g* for 2 min to remove any residual solutions contained in the filter. Supernatants were analyzed by MALDI-MS according to the manufacturer’s instruction.

### MALDI-MS measurement

MALDI-MS measurements were performed in positive linear mode using MALDI-8020 RUO (Shimadzu Corporation, Kyoto, Japan) equipped with a 200 Hz Nd : YAG laser (355 nm). Mass calibration was performed using 6 peaks with *m/z* 4365.4, 5381.4, 6411.6, 7274.0, 8369.8, and 10300.1 from *Escherichia coli* DH5α. Five individual mass spectra were acquired for each fungal extract in the range from *m/z* 2,000 to 20,000. Biomarker search and peak matching were carried out using eMSTAT Solution™ software (Shimadzu Corp., Kyoto, Japan).

## Result

### Fungal isolation and drug susceptibility test

**Figure 1:**
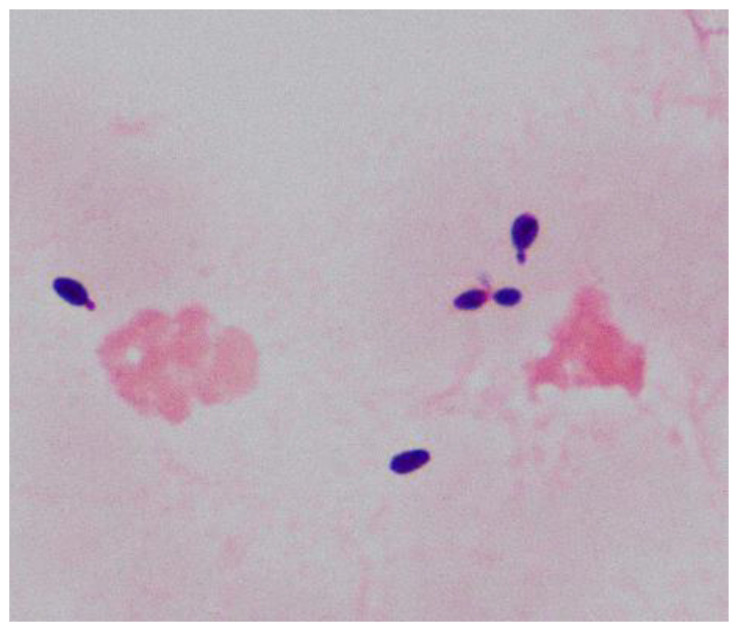
Morphology of yeast-like cells by Gram-staining (1000x) from the positive blood culture.

**Figure 2:**
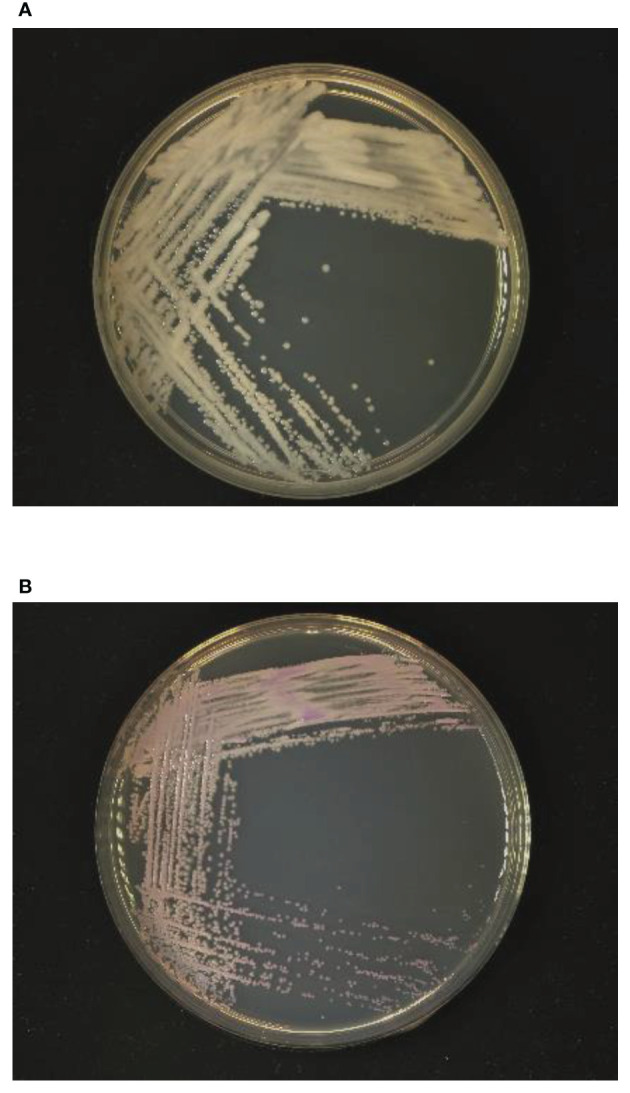
Growth of yeast-like colonies after 48h incubation in **(A)** Sabouraud dextrose agar and **(B)** CHROM agar Candida medium.

Samples of the positive blood culture were Gram stained and revealed the presence of yeast-like cells (**Figure 1**). The positive blood culture samples were inoculated on Sabouraud Dextrose Agar (SDA) plate, Potato Dextrose Agar (PDA), and CHROM agar Candida medium at 35°C. After 48h of incubation, a large number of monomorphic, cream-colored smooth colonies were observed on the SDA and PDA. Purple-colored colonies were observed on the CHROM agar Candida medium (**Figure 2**). The smear of the colony showed yeasts that were observed by the direct smear of the blood culture specimen. The isolate was identified as *Candida* species. The MICs of the isolate to fluconazole and micafungin were 4 μg/mL and 0.5 μg/mL, respectively (**Table 1**). According to the breakpoints of fluconazole and micafungin in *Candida* species, the isolate showed intermediate resistance.

### Fungal identification, virulence, and drug-resistance

The ITS sequence of the isolate was 98.8% identical to the sequence of *W. tropicalis* (GenBank accession number MN218416). Therefore, the isolate was confirmed to be *W. tropicalis* and designated as *W. tropicalis* JUWT001.

The *W. tropicalis* JUWT001 harbored two virulence genes encoding protein kinase regulators, *IRE1* and *CBK1*, that cause pathogenic fungal infections (Talapko et al., 2021). The IRE1 protein had 69.4% and 94.5% identity to that in *C. albicans* SC 5314 (GenBank accession no. GCF_000182965) and *W. sorbophila* NBRC 1583^T^ (accession no. GCF_002251995), respectively. The CBK1 protein had 40.3% and 90.7% identity to that in *C. albicans* SC 5314 (accession no. GCF_000182965) and *W. sorbophila* NBRC 1583^T^ (accession no. GCF_002251995), respectively.

**Figure 3:**
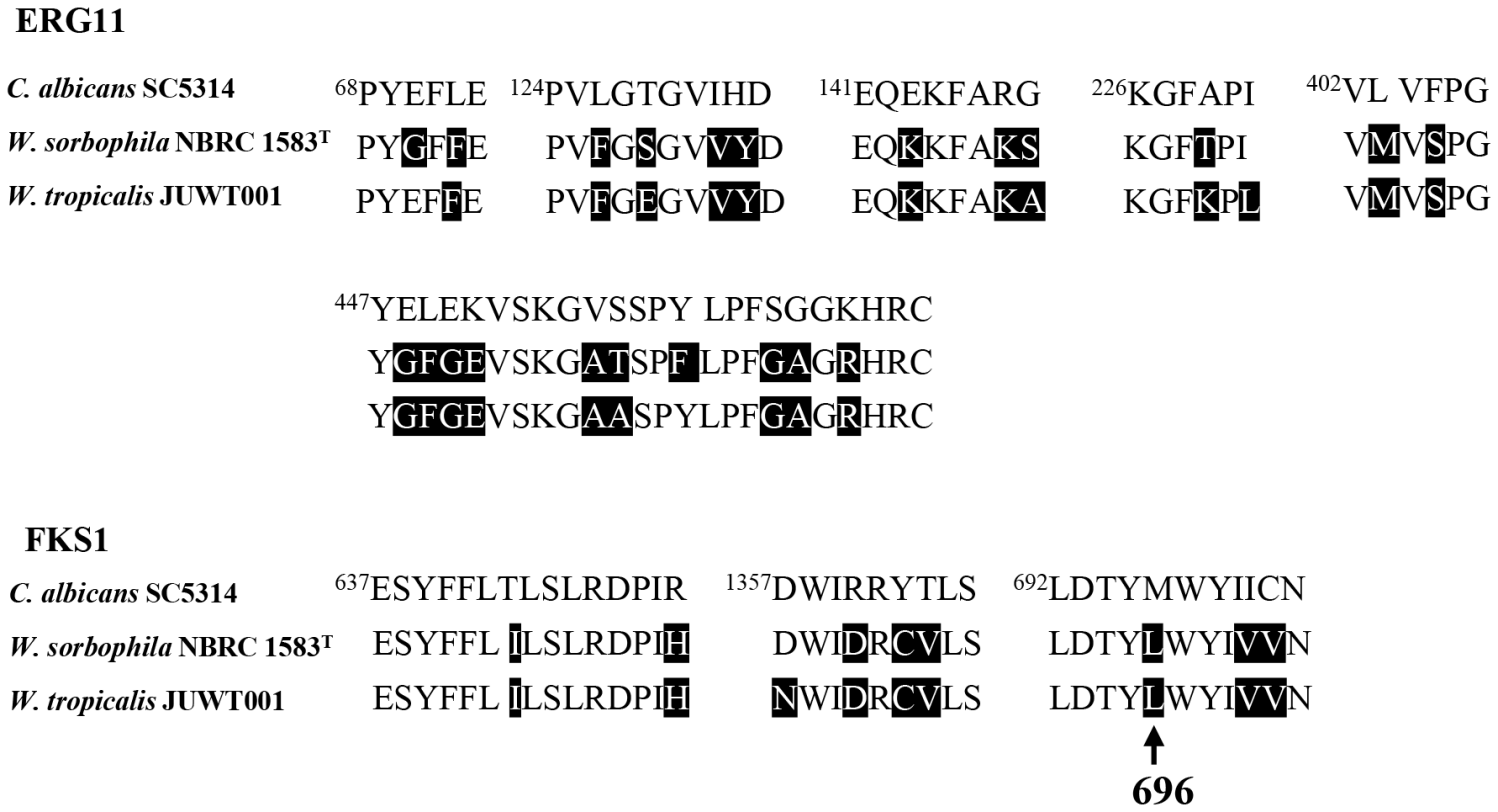
Amino acid sequence alignments of hot spot regions of ERG11 and FKS1 in *C. albicans* SC 5314 (GenBank accession no. GCF_000182965), *W. sorbophila* NBRC 1583^T^ (accession no. GCF_002251995) and *W. tropicalis* JUWT001. Amino acid substitutions are shaded in black, compared with *C. albicans* SC 5314. The arrow indicates an amino acid substitution of FKS1 from Met to Leu at the position of 696.

The isolate had amino acid substitutions in ERG11 and FKS1 associated with azole and echinocandin resistance, respectively. Twenty-one amino-acid substitutions in ERG11 and 9 amino-acid substitutions in FKS1 were detected in hot spot regions which were highly conserved antimicrobial-binding domain in fungi (Favre et al., 1999; Papp et al., 2018) (**Figure 3**). Of the nine amino acid substitutions of FKS1, an amino acid substitution from Met to Leu at the 696 position was reported in *C. parapsilosis* in 2018 (Papp et al., 2018; Spettel et al., 2019). There is no report on the remaining mutations in these resistant factors in the hot spot regions (**Figure 3**).

### Phylogenic analysis

**Figure 4:**
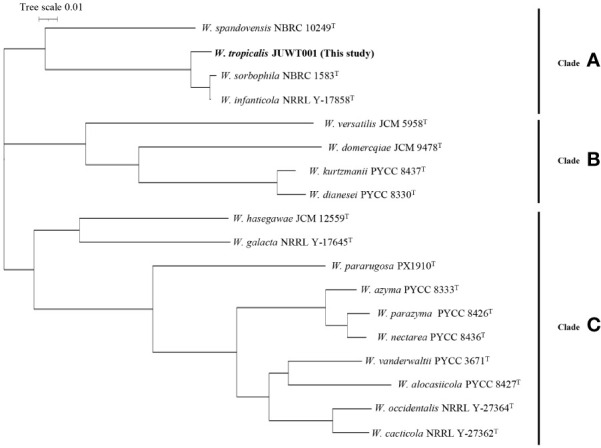
Phylogenetic tree of a clinical isolate and 17 type strains of *Wickerhamiella* species. The phylogenetic tree revealed three clades: Clades A, B, and C.

As shown in **Figure 4**, the phylogenetic tree revealed three clades: A, B, and C. *W. tropicalis* JUWT001 belonged to clade A, along with *W. sorbophila* (GenBank accession no. GCA_023629035) and *W. infanticola* (accession no. GCA_004125145), which have already been reported to be isolated from humans. Another previously reported clinical isolate, *W. pararugosa* (accession no. JAMAJR000000000), belonged to clade C. The other species were mostly isolated from plant (Lachance et al., 1998; Nakase et al., 2007; Barata et al., 2008; Wang et al., 2008; Lachance et al., 2010; de Vega et al., 2017).

### MALDI-MS analysis

**Figure 5:**
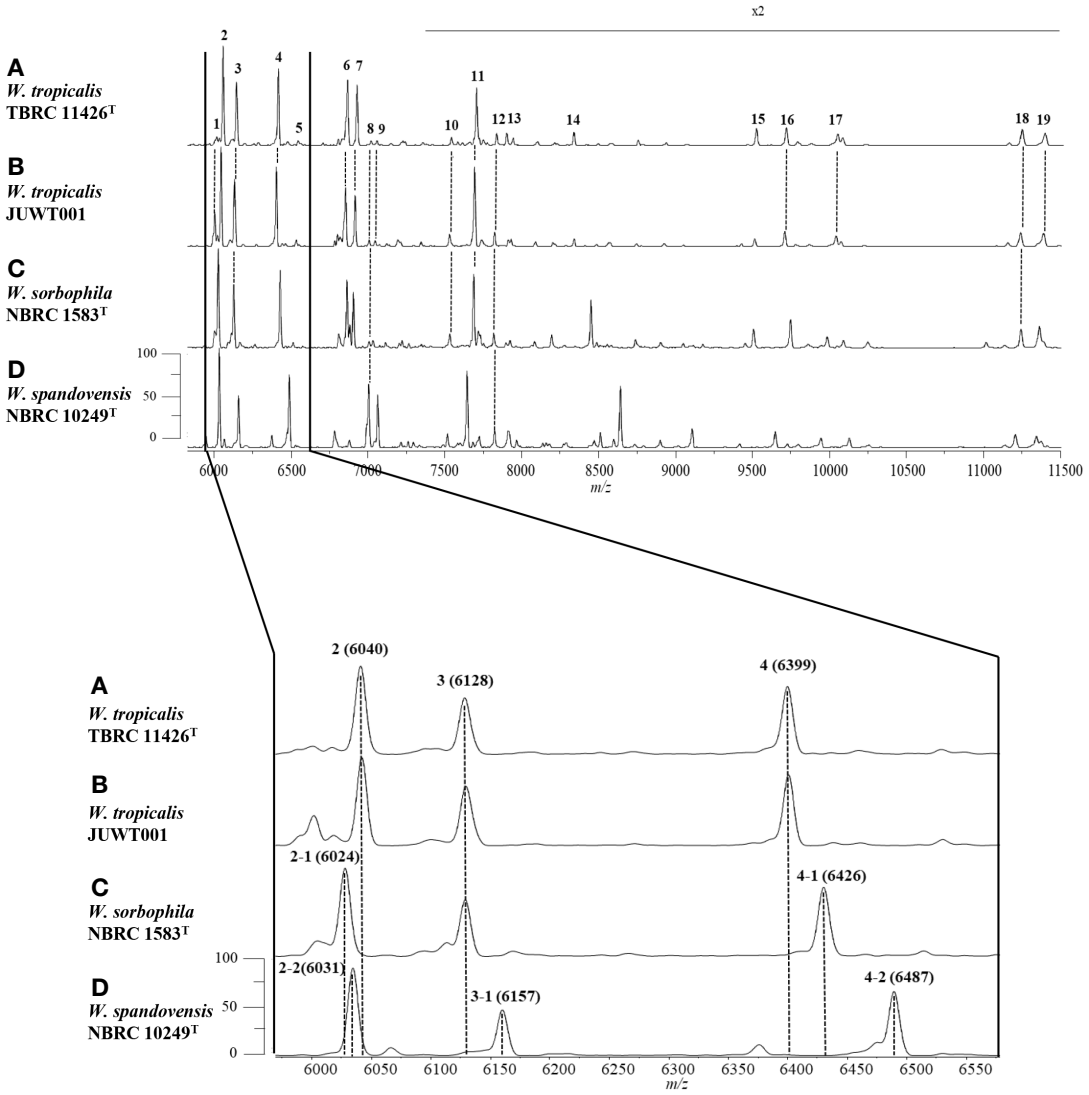
Representative mass spectra of **(A)** *W. tropicalis* TBRC 11426^T^, **(B)** JUWT001, **(C)** *W. sorbophila* NBRC 1583^T^, and **(D)** *W. spandovensis* NBRC 10249^T^ in the protein fractions. Upper figure indicates mass spectra from *m/z* 6,000 to 11,500. Peak numbers correspond to those detailed in **Table 2**. Amplification of the *m/z* 6,000 to 6600 section of mass spectra results (lower figure) reveals small variations of *m/z* values for peaks 2, 3 and 4 for *W. sorbophila* and *W. spandovensis*, with reference to those of *W. tropicalis* strains, as detailed in **Table 2**.

We investigated the feasibility in identifying *W. tropicalis* using MALDI-MS. The protein fraction and cell lysate mass spectra of *W. tropicalis* JUWT001 were examined by MALDI-MS along with the type strains of *W. tropicalis*, *W. sorbophila*, and *W. spandovensis.* The numbers of mass peaks detected in the protein fractions were higher than in the cell lysates for all strains tested (**Supplementary Figure 1**). Therefore, the protein fractions were used in the following study. As shown in **Figure 5**, we observed that the protein fraction mass spectra of *W. tropicalis* JUWT001 were almost identical to *W. tropicalis* type strain, but different from *W. sorbophila* and *W. spandovensis* type strains. In order to find useful biomarker peaks, we used the eMSTAT Solution software. Biomarker peaks were selected from those with a relative peak intensity ratio of 0.5% or higher which was detected in all five replicates. The number of major peaks were 19 for the *W. tropicalis* type strain, 18 for *W. tropicalis* JUWT001, 17 for *W. sorbophila* type strain and 16 for *W. spandovensis* type strain (data not shown). The 19 peaks of the *W. tropicalis* type strain were then used as biomarker peaks, and the matching peaks of the observed masses to the biomarker masses were judged from a tolerance within 500 ppm. Then, the homology to the reference peaks of *W. tropicalis* type strain were evaluated for *W. tropicalis* JUWT001, *W. sorbophila* type strain, and *W. spandovensis* type strain (**Figure 5**). As shown in **Table 2**, 15 mass peaks (79%) of *W. tropicalis* JUWT001, 6 mass peaks (32%) of *W. sorbophila* type strain, and 2 mass peaks (11%) of *W. spandovensis* type strain were detected at the same mass as the 19 mass peaks of *W. tropicalis* type strain. Compared with the *W. tropicalis* type strain and JUWT001, *W. sorbophila* and *W. spandovensis* type strain had unique peaks shown in **Figure 5** and **Table 2**. The same peak (less than 500 ppm) at *m/z* 6,040 (peak No. 2) were observed in *W. tropicalis* JUWT001 and *W. tropicalis* type strain, whereas the different peaks at *m/z* 6,024 (peak No. 2-1) and *m/z* 6,031 (peak No. 2-2) were observed in *W. sorbophila* and *W. spandovensis* type strains, respectively. The same peaks at *m/z* 6,128 (peak No. 3) were observed in *W. tropicalis* and *W. sorbophila*, whereas the different peaks of *m/z* 6,157 (peak number 3-1) was observed in *W. spandovensis*. The same peaks at *m/z* 6,399 (Peak No. 4) were observed in *W. tropicalis*, whereas the different peaks at *m/z* 6,426 (peak No. 4-1) and *m/z* 6,487 (No. 4-2) were observed in *W. sorbophila* and *W. spandovensis*, respectively. These specific peaks were observed not only in the protein fraction, but also in the mass spectra of cell lysates cultured on SDA, PDA, and CHROM agar Candida medium (data not shown).

**Table 2:** Characteristics of the peaks of the protein fractions in the MALDI-MS analysis in the isolates of *Wickerhamiella* species.

Strains		Peak No																		
		1	2	3	4	5	6	7	8	9	10	11	12	13	14	15	16	17	18	19
*W. tropicalis* TBRC 11426^T^	Average *m/z*	6000	6040	6128	6399	6529	6849	6911	7002	7040	7524	7688	7817	7885	8321	9507	9702	10035	11234	11383
	SE	0.47	0.28	0.33	0.27	0.26	0.33	0.33	0.40	0.51	0.34	0.34	0.32	0.32	0.48	0.33	0.30	0.31	0.59	0.46
*W. tropicalis* JUWT001	Average *m/z*	6003	60.43	6131	6402	NA	6853	6914	7006	7044	7528	7691	7821	NA	NA	NA	9707	10040	11239	11389
	SE	0.35	0.31	0.33	0.32	NA	0.39	0.35	0.45	0.51	0.42	0.45	0.53	NA	NA	NA	0.52	0.63	0.84	0.60
*W. sorbophila* NBRC 1583^T^	Average *m/z*	NA	6024^a^	6125	6426^d^	NA	NA	NA	7004	NA	7527	7684	7814	NA	NA	NA	NA	NA	11240	NA
	SE	NA	0.21	0.28	0.32	NA	NA	NA	0.46	NA	0.63	0.41	0.41	NA	NA	NA	NA	NA	0.39	NA
*W. spandovensis* NBRC10249^T^	Average *m/z*	NA	6031^b^	6157^c^	6487^e^	NA	NA	NA	7003	NA	NA	NA	7821	NA	NA	NA	NA	NA	NA	NA
	SE	NA	0.36	0.34	0.40	NA	NA	NA	0.41	NA	NA	NA	0.52	NA	NA	NA	NA	NA	NA	NA

m/z, mass to charge ratio; SE, standard error; NA, not assigned.

a Peak No. 2-1 (specific peak of *W. sorbophila*) in **Figure 5**.

b Peak No. 2-2 (specific peak of *W. spandovensis*) in **Figure 5**.

c Peak No. 3-1 (specific peak of *W. spandovensis*) in **Figure 5**.

d Peak No. 4-1 (specific peak of *W. sorbophila*) in **Figure 5**.

e Peak No. 4-2 (specific peak of *W. spandovensis*) in **Figure 5**.

## Discussion

To date, *W. tropicalis* strains were only isolated from a grease trap obtained in Thailand in 2017 and from sea surface microlayer in Taiwan in 2005 (Sakpuntoon et al., 2020). To our knowledge, this is the first report that *W. tropicalis* can cause human infectious disease.

The immunocompromised patients have possibilities of severe infections caused by environmental fungi including *Wickerhamiella* species. The mortality of non-*Candida albicans* species in pediatric patients with malignancies was 26.3% between 2015 and 2019 (Vasileiou et al., 2020). The previous studies reported that the 5 patients with *W. pararugosa* bloodstream infections were immunocompromised because of malignancy, sarcoma, surgery, and leukemia, and 2 of the 5 patients could not be cured (Nasri et al., 2023). Similarly for *W. tropicalis* infections, accurate and rapid diagnosis of infection in immunocompromised patients is critical.

*Wickerhamialla* species show intrinsically intermediate resistance/resistance to fluconazole. *W. pararugosa* clinical isolates causing bloodstream infections had MICs of 4 to 8 μg/mL against fluconazole (Taj-Aldeen et al., 2014; El Helou and Palavecino, 2017; Noni et al., 2020; Nasri et al., 2023). *W. infanticola* isolate from environmental sample had MICs of 4 μg/mL against fluconazole (Maciel et al., 2019). However, there are no MIC breakpoints for azole and echinocandin in *Wickerhamiella* species, and the breakpoints for *Candida* species in CLSI are used in clinical laboratories in many countries. Therefore, antifungal agents should be used with caution in the treatment of *Wickerhamiella* infections.

Furthermore, the identification of non-*Candida* species is important for initiating early effective antifungal therapy because these species, such as the *Cryptococcus* and *Wickerhamiella* species, have the potential to develop antifungal resistance. Previous studies on the identification of yeast isolates using MALDI-MS have shown that the identification accuracy of yeasts by MALDI-MS is not sufficient, with an identification accuracy of 96.8% for *Candida* species and 84.2% for non-*Candida* species (Zhang et al., 2014). On the other hand, the identification accuracy of *Cryptococcus* species by MALDI-MS was reported to have improved from 76.0% using a commercial database, to 100% by detecting five new biomarker peaks (Zvezdanova et al., 2020). Thus, it is essential for clinical laboratories to establish biomarker peaks and fingerprinting libraries for rare yeast species, including *Wickerhamiella* and *Cryptococcus*. The three MALDI-MS peaks specific for *W. tropicalis* obtained in this study may serve as an indicator for the identification of rare fungal species, including *Wickerhamiella* species, in routine microbiological examinations.

This study has the following limitations. First, this study was conducted at a single institution. The results should be evaluated and discussed at other study sites in the future. Second, only one sample was obtained in this study because *W. tropicalis* is rare to obtain from humans. Further data on clinical isolates of *Wickerhamiella* species should be collected. Third, the lack of genomic data on *W. tropicalis* precluded the use of theoretical protein masses in this study. It is hoped that genomic data will be constructed in the future.

In conclusion, MALDI-MS analysis has the potential for rapid identification of *Wickerhamiella* species as well as other fungus infections. It is important to establish the database using MALDI-MS for identification of yeast-like fungi causing severe infections in clinical laboratories.

## Data availability statement

The datasets presented in this study can be found in online repositories. The names of the repository/repositories and accession number(s) can be found in the article/**Supplementary Material**.

## Ethics statement

The studies involving humans were approved by the Ethical Committee of Juntendo University. The studies were conducted in accordance with the local legislation and institutional requirements. The human samples used in this study were acquired from a by- product of routine care or industry. Written informed consent for participation was not required from the participants or the participants’ legal guardians/next of kin in accordance with the national legislation and institutional requirements. Written informed consent was obtained from the minor(s)’ legal guardian/next of kin for the publication of any potentially identifiable images or data included in this article.

## Author contributions

ST: Conceptualization, Data curation, Formal Analysis, Funding acquisition, Investigation, Methodology, Validation, Visualization, Writing – original draft, Writing – review & editing. KT: Methodology, Visualization, Writing – original draft, Writing – review & editing. JF: Resources, Writing – original draft. MF: Resources, Writing – original draft. MS: Resources, Writing – original draft. YF: Resources, Writing – original draft. YS: Writing – original draft. TaK: Resources, Writing – original draft. MC: Resources, Writing – original draft. MW: Resources, Writing – original draft. YH: Writing – original draft. TM: Writing – original draft. TN: Writing – original draft. TeK: Writing – original draft. TT: Conceptualization, Funding acquisition, Project administration, Supervision, Visualization, Writing – original draft, Writing – review & editing. YT: Project administration, Visualization, Writing – original draft, Writing – review & editing.
