# Supplementary material for: Isolation and identification of Wickerhamiella tropicalis from blood culture by MALDI-MS
Source: Front Cell Infect Microbiol. 2024 Mar 6;14:1361432. doi: 10.3389/fcimb.2024.1361432 (PMC10953818; doi:10.3389/fcimb.2024.1361432)
Supplement: Supplementary file 1 [file Image_1.pdf]

Figure S1

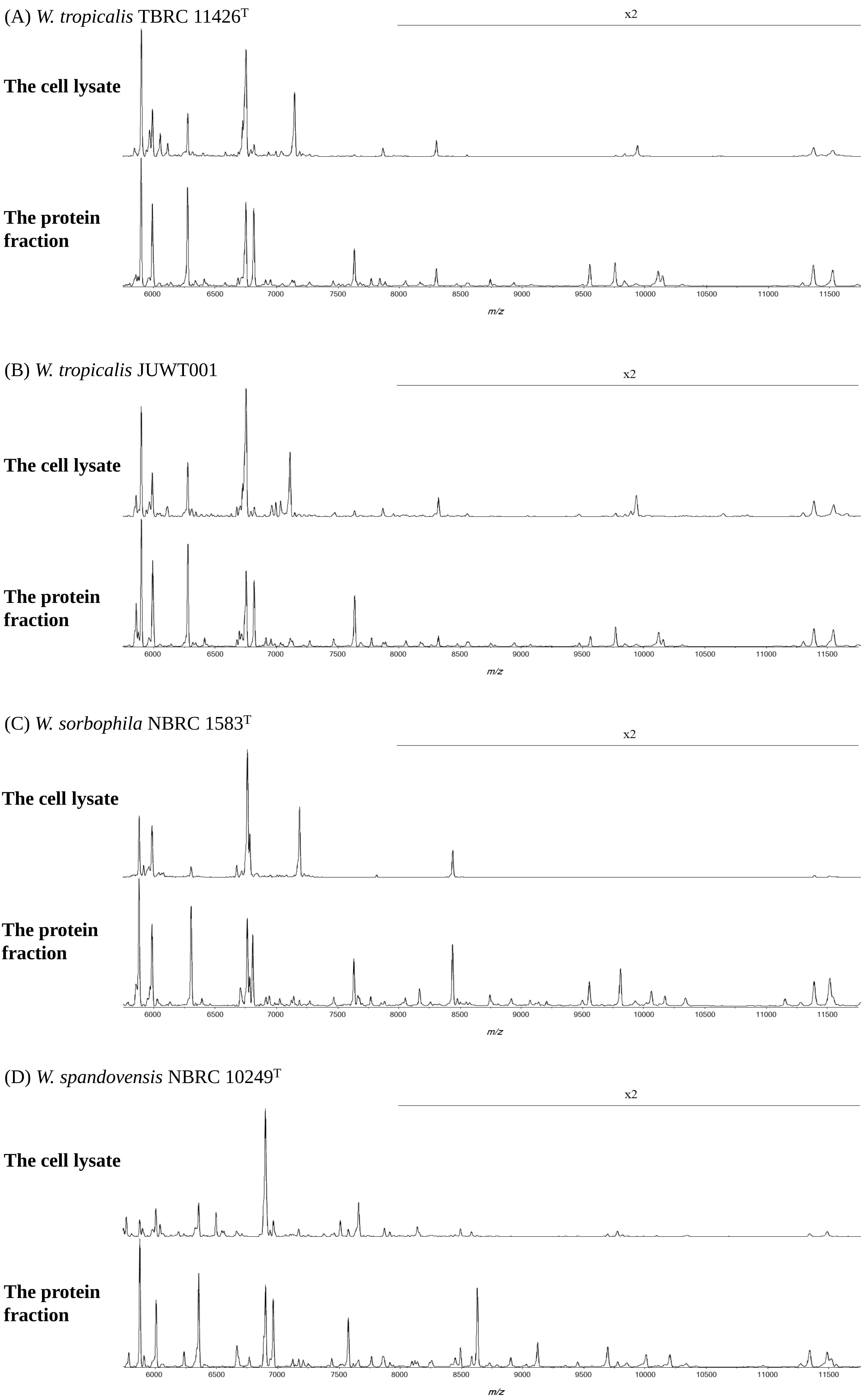

Supplementary Figure 1: Representative mass spectrums of the cell lysates and the protein fractions of (A) *W. tropicalis* TBRC 11426^T, (B) JUWT001, (C) *W. sorbophila* NBRC 1583^T, and (D) *W. spandovensis* NBRC 10249^T from *m/z* 6,000 to 11,500.
